# A “Coiled-Coil” Motif Is Important for Oligomerization and DNA Binding Properties of Human Cytomegalovirus Protein UL77

**DOI:** 10.1371/journal.pone.0025115

**Published:** 2011-10-05

**Authors:** Christina Sylvia Meissner, Pánja Köppen-Rung, Alexandra Dittmer, Sara Lapp, Elke Bogner

**Affiliations:** Institute of Virology, Charité Universitätsmedizin Berlin, Berlin, Germany; Queen's University, Canada

## Abstract

Human cytomegalovirus (HCMV) UL77 gene encodes the essential protein UL77, its function is characterized in the present study. Immunoprecipitation identified monomeric and oligomeric pUL77 in HCMV infected cells. Immunostaining of purified virions and subviral fractions showed that pUL77 is a structural protein associated with capsids. *In silico* analysis revealed the presence of a coiled-coil motif (CCM) at the N-terminus of pUL77. Chemical cross-linking of either wild-type pUL77 or CCM deletion mutant (pUL77ΔCCM) implicated that CCM is critical for oligomerization of pUL77. Furthermore, co-immunoprecipitations of infected and transfected cells demonstrated that pUL77 interacts with the capsid-associated DNA packaging motor components, pUL56 and pUL104, as well as the major capsid protein. The ability of pUL77 to bind dsDNA was shown by an *in vitro* assay. Binding to certain DNA was further confirmed by an assay using biotinylated 36-, 250-, 500-, 1000-meric dsDNA and 966-meric HCMV-specific dsDNA designed for this study. The binding efficiency (BE) was determined by image processing program defining values above 1.0 as positive. While the BE of the pUL56 binding to the 36-mer bio-pac1 containing a packaging signal was 10.0±0.63, the one for pUL77 was only 0.2±0.03. In contrast to this observation the BE of pUL77 binding to bio-500 bp or bio-1000 bp was 2.2±0.41 and 4.9±0.71, respectively. By using pUL77ΔCCM it was demonstrated that this protein could not bind to dsDNA. These data indicated that pUL77 (i) could form homodimers, (ii) CCM of pUL77 is crucial for oligomerization and (iii) could bind to dsDNA in a sequence independent manner.

## Introduction

The assembly of most double-stranded tailed bacteriophages and herpesviruses is a common, multistep process during viral maturation. The concatemeric, newly synthesized DNA has to be cleaved into unit-length genomes prior to packaging into empty capsids and condensed into a structure of near crystalline density. Enzymes involved in this process are responsible for site-specific cleavage and insertion of the DNA into the procapsid [1], [2]. These enzymes known as terminases power the packaging via their ATPase activity. In previous studies we have demonstrated that the HCMV terminase consists of two subunits, the large one encoding pUL56 and the small one pUL89 [3]–[6], whereas each subunit has a different function. HCMV pUL56 is required for recognition and binding to DNA at specific sequence motifs (packaging signals, e.g. pac1 and pac2), it mediates interaction of the DNA-protein-complex with the portal protein pUL104 and catalyzes the import of one unit-length genome into the capsid by providing ATP [5]–[8]. The small subunit pUL89 is required for the completion of the packaging process by cleavage of concatemeric DNA into unit-length genomes (two strand nicking; 9). The terminase together with the portal protein pUL104 [10], [11] form the molecular nanomotor which enables the insertion of the viral genome into capsids against growing internal forces [12]. Smith et al. (2001) have demonstrated that forces during packaging of bacteriophage phi can increase to 57 pico Newton (pN), thus representing one of the strongest biological nanomotors [13].

In addition to the terminase subunits and the portal protein Borst et al. (2007) demonstrated that HCMV pUL52 is another essential protein for cleavage and packaging [14]. It is hypothesized that pUL52 may be involved in the closing procedure of the capsid after the DNA is encapsidated. However, it is most likely that several additional viral proteins are required for the complex DNA packaging process. One candidate is the protein UL77 (pUL77), a conserved core gene of HCMV. Analysis of deletion mutants provided evidence that this protein is essential for HCMV replication in fibroblasts [15], [16]. The herpes simplex virus type 1 (HSV-1) homolog, pUL25, is part of a so-called capsid-vertex-specific component (CVSC), an elongated molecule that is localized on the outer surface of capsids whereas five copies surround the capsid vertices [17].The protein has been shown to be situated at multiple sites on the surface of capsids adjacent to the pentons at the vertices [18], [19]. Further analysis revealed that pUL25 of pseudorabies virus (PRV) and HSV-1 is required for nuclear egress of C-capsids [18], [20], [21] as well as for uncoating of the viral genome after penetration of the host cell [22]. However, the function of HCMV pUL77 is so far unknown.

To investigate its role in DNA packaging pUL77 (i) was identified in infected cells and extracellular virions, also (ii) its interactions with viral DNA packaging proteins, a prerequisite of its proposed function, and (iii) DNA binding abilities were analysed. By *in silico* analysis a coiled-coil motif (CCM) at the N-terminus of pUL77 was identified. Its role in oligomerization as well as DNA binding was confirmed using a CCM-deletion mutant pUL77ΔCCM in *in vitro* studies.

## Methods

### Cells and virus

Human foreskin fibroblasts (HFF; PromoCell, Germany) and human embryonic kidney (HEK) 293T cells (ATTC, USA) were grown in Dulbecco's minimal essential medium (DMEM) supplemented with 10% fetal calf serum (FCS), 2 mM glutamine, penicillin (5 U/ml), and streptomycin (50 µg/ml). HFF cells at passages 10 to 15 were used for infections and experiments were carried out with confluent cell monolayers (1.5×10^7^ cells). Infection of HFF with HCMV AD169 at a MOI of 3 was carried out as described before [4].

### Plasmid construction and oligonucleotides

Restriction enzymes were purchased from Fermentas (St. Leon-Rot, Germany) and used as instructed by the manufacturer. The UL77 gene was generated by PCR amplification from cosmid pCM1075 [23] using the following pair of oligonucleotides (restriction sites for EcoRI and XbaI or XhoI were underlined):

5′-CGGAATTCTGATGAGTCTGTTGCACACCTTTTGGCGG-3′ and

5′-TCGGCTCGAGTTACAACACCGCCACGCTCGG-3′ for pGEX-UL77

or

5′-GCGAATTCTGATGAGTCTGTTGCACACCTTTTGGCGG-3′ and

5′-GCTCTAGATTACAACACCGCCACGCTCGG-3′ for myc-UL77 and pcDNA-UL77

or

5′- CAGAATTCATGCTCGACGAAGGACCGTCG-3′ and

5′- CATCTAGATTACAACACCGCCACG TCGG-3′ for pcDNA-UL77ΔCCM

The PCR product as well as the vector pGEX-6P-1, pcDNA3.1 Myc and pcDNA3.1/His C (Invitrogen, Karlsruhe, Germany), were separately digested with EcoRI and XbaI or XhoI, and the PCR product was ligated in-frame into the vectors yielding pGEX-UL77, myc-UL77, pcDNA-UL77 and pcDNA-UL77ΔCCM. Constructs pcDNA-UL56 [18] and pcDNA-UL104 [14] were designed for previous studies. Plasmid pHM123 encoding IE1 is described by Plachter et al. [24].

### Purification of GST-UL77 and PreScission treatment

A fresh overnight culture of E. coli BL21 carrying the plasmid pGEX-UL77 was grown in LB media. After the cells reached an OD_600_ of 0.5 the GST-protein expression was induced by addition of isopropyl-1-thio-ß-D-galactopyranoside (IPTG) to a final concentration of 0.1 mM and incubated for 2 h at 37°C. Sedimented cells were lysed in 40 ml binding buffer (20 mM sodium phosphate, 0.15 M NaCl, pH 7.4) containing 250 µM MgCl_2_, 25 µM MnCl_2_, 400 µg DNase I and 4 mg lysozyme, incubated for 30 min and sonicated on ice. Undissolved material was sedimented at 5.000× g at 4°C and supernatant passed through a 0.2 µm filter. The purification was performed with a GSTrap™ column (1 ml bed volume) by using an ÄKTA FPLC (GE Healthcare, Freiburg, Germany) according to the instruction of the manufacturer. The column was washed with three bed volumes binding buffer prior to loading the proteins.

Protein is eluted from the column with 10 bed volumes of elution buffer (50 mM Tris-HCl, 10 mM reduced glutathione, pH 8.0) and used in cross-linking experiments and *in vitro* binding assays.

For usage as antigen in antibody purification GST-tag needs to be removed. To cleave the GST tag from GST-UL77, the bound protein was incubated with 80 units of PreScission protease (GE Healthcare, Freiburg, Germany) for 12 h at 4°C. After removal of the GST tag the resulting protein was eluted in 10 fractions (1 ml) of PreScission buffer (50 mM Tris-HCl pH 7.0, 150 mM NaCl, 1 mM EDTA, 1 mM DTT). The fractions containing the protein rpUL77 were stored at −80°C.

### Antibody against pUL77

**Figure 1:**
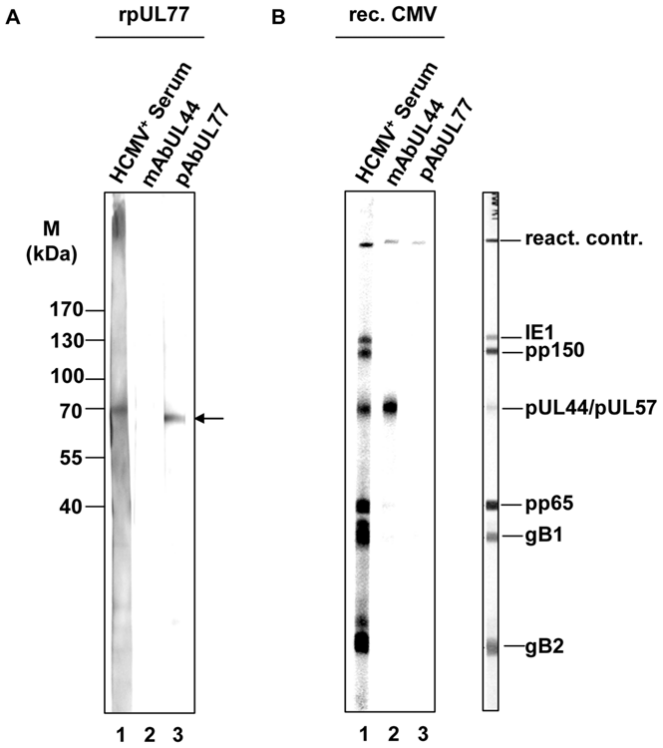
Characterization of the antibody pAbUL77. (A) Purified GST-UL77 was separated by 10% SDS-PAGE and transferred to nitrocellulose. Immunoblot strips were reacted with hyper immune serum Cytotect (HCMV^+^ Serum, lane 1), anti-pUL44 (mAbUL44, lane 2) and pAbUL77 (lane 3). (B) Immunoblot stripes containing recombinant proteins encoding immunodominant epitopes of IE1 (53 kDa), pp150 (50 kDa), pUL44/pUL57 (45 kDa), pp65 (31 kDa), gB1 (25 KDa) and gB2 (18 kDa) were reacted with hyper immune serum (HCMV^+^ Serum, lane 1), anti-pUL44 (mAbUL44, lane 2) and pAbUL77 (lane 3). Control stripe incubated with a weak HCMV+ serum from Mikrogen Diagnostics was shown on the right. Arrows on the right side indicate the position of pUL77. Molecular weight markers (M) are shown on the left side.

HCMV pUL77 specific human polyclonal antibody pAbUL77 was purified from high titer human serum (IgG positive patient serum selected by CMV diagnostic) by column affinity chromatography (Affigel 10/15-pUL77). Affinity purification was performed as described previously [11]. The specificity of the purified pAbUL77 was determined using immunoblots with rpUL77. Immunoblot strips were reacted with Cytotect (Biotest, Germany), a hyper immune serum against HCMV (HCMV^+^, 1∶500; Fig. 1A, lane 1), CMV pp52 (CH16; Santa Cruz Biotechnology, Heidelberg, Germany) monoclonal antibody against pUL44 (mAbUL44; Fig. 1A, lane 2) and pAbUL77 (Fig. 1A, lane 3). Only Cytotect and the purified antibody pAbUL77 reacted on strips with rpUL77. As an additional control for specificity of pAbUL77 immunoblot analyses with different viral proteins were performed. Immunoblot stripes (kindly provided by Mikrogen Diagnostic, Neuried, Germany) containing recombinant polypeptides encoding immediate early protein 1 (IE1; 53 kDa), tegument protein pp150 (50 kDa), processivity factor pUL44 and single stranded binding protein pUL57 (45 kDa), tegument protein pp65 (31 kDa), glycoprotein B (gB1, 25 kDa; gB2, 18 kDa) only reacted with reconvalsecent serum (Fig. 1B, lane 1) and specific antibody against pUL44 (Fig. 1B, lane2). These observations demonstrate that pAbUL77 is a monospecific antibody against pUL77.

### Isolation and fragmentation of HCMV extracellular virions

Four days after infection extracellular virions were separated from dense bodies and non-infectious enveloped particles (NIEPs) by sedimentation through a sodium-tartrate gradient according to Talbot and Almeida [25]. Isolated virions were incubated with non-ionic detergent (1% (v/v) NP-40) for 10 min on ice prior to separation through a 15% (w/v) sucrose cushion in TE buffer (100.000× *g*, 1 h at 4°C). The supernatant contains the envelope fraction. The sediment which constitutes the capsid/tegument fraction was treated with 1% (v/v) ß-mercaptoethanol for 10 min on ice. Following this treatment, the capsid/tegument fraction was loaded onto a 15% (w/v) sucrose cushion and sedimented (100.000× *g*, 1 h at 4°C). The supernatant contains the tegument and the sediment the capsid fraction. The tegument as well as the envelope fraction were precipitated by 10% (v/v) TCA and dissolved in TE buffer. The capsid, tegument and envelope fraction as well as mock-, infected cells and extracellular virions were subjected to SDS-PAGE prior to immunostaining.

### Immunoprecipitation

For immunoprecipitation HCMV AD169 infected or mock-infected cells (T-75 cm^2^ flasks; 2×10^6^ cells) were 60 h post infection (p.i.) radiolabeled with 50 µCi/ml [^35^S] methionine for 12 h. Total cell extracts were prepared from labelled cultures by solubilisation in immunoprecipitation buffer (20 mM Tris-HCl pH 7.5, 100 mM NaCl, 1% NP-40, 5 mM EDTA, 25 mM iodacetamide, 0.4% sodiumdeoxycholate, 1 mM PMSF, 100 U/ml Trasylol) and ultrasonic treatment. Insoluble material was sedimented for 30 min at 100.000× g and 4°C. Comparable amounts of extracts and pUL77-specific antibody pAbUL77 (1∶5) were used for precipitation prior to SDS-PAGE and autoradiography [6].

For co-immunoprecipitation either HFF cells (2×10^6^) were infected with HCMV AD169 (MOI 2) or 293T cells (2×10^6^) were transfected with (i) myc-UL77 and pcDNA-56, (ii) myc-UL77 and pcDNA-MCP , (iii) myc-UL77 and pcDNA-UL104, (iv) myc-UL77 and pHM123 (IE1). Total cell extracts were prepared 72 h after infection or 48 h after transfection by solubilisation in co-immunoprecipitation buffer (20 mM Tris-HCl pH 7.5, 150 mM NaCl, 0.5% Tween, 5 mM EDTA) containing the protease inhibitor mix M (Serva, Heidelberg, Germany) prior to ultrasonic treatment. Insoluble material was sedimented for 10 min at 100.000× *g*. Comparable amounts of extracts were used for precipitation. For mock-infected or infected cells pAbUL77 (1∶10) as well as specific antibody pAbUL56 specific for pUL56 [5], pAbUL104 specific for pUL104 [11], mAb63-27 specific for IE1 and mAb28-4 specific for the major capsid protein (MCP; 1∶10) were used for precipitation from both sides.

To analyse if protein- protein interactions are observed with solitary expressed proteins, pUL77 was precipitated using specific Myc-Tag antibody (1∶500; Cell Signaling) in co- transfection experiments. Exact procedure was described previously [6]. Immunoprecipitates were analyzed by SDS-PAGE followed by immunoblotting.

### PAGE and immunoblot analysis

Extracts from mock-infected, infected cells or immunoprecipitates were solubilzed in 4× sample buffer [4% (v/v) ß-mercaptoethanol, 0.01% (w/v) bromphenol blue, 4% (w/v) glycerol, 4% (w/v) SDS, 0.2 M Tris-HCl (pH 6.8)] prior to separation on 8 or 10% (w/v) sodium dodecyl sulfate-polyacrylamide gel. Proteins were transferred to nitrocellulose sheets and subjected to immunoblot analysis as described previously [26]. The antibody pAbUL77, pAbUL56 specific for pUL56 [5], pAbUL104 specific for pUL104 (14), mAb63-27 specific for IE1 and mAb28-4 specific for the major capsid protein (MCP; 1∶10), were used as the primary antibodies. To analyze purified extracellular virions and the separated capsid fractions, pAbUL77, CMV pp28 (5C3; (1∶500;Santa Cruz Biotechnology, INC., Heidelberg, Germany) specific for the tegument protein pp28, mAb27-156 (1∶10) specific for the glycoprotein B and mAb28-4, served as primary antibodies, respectively.

For detection of primary antibody binding, horseradish peroxidase-conjugated anti-human or anti-mouse F(ab′)_2_ fragments (1∶5000 in PBS with 0.3% BSA; Abcam, Cambridge, United Kingdom) and the ECL (Super Signal West Pico) reagent were used as recommended by the supplier (Pierce; Thermo Fisher Scientific, Germany).

### In vitro translation

The plasmids pcDNA-UL77, pcDNA-UL77ΔCCM, pcDNA-UL56 or pcDNA-MCP (1 µg each) were incubated with [^35^S] methionine/cystein (10 mCi/ml) and 40 µl TNT® T7 Quick Master Mix (Promega, Mannheim, Germany) in a final volume of 50 µl for 1.5 h at 30°C. Translation products were analyzed by 8% SDS-PAGE or subjected to DNA binding assay without any sedimentation step.

### Nuclear extracts for DNA binding analysis

For preparation of cells extracts for DNA binding analysis HFF cells (1×10^7^) were infected with HCMV AD169 (MOI 5). The method used for extraction has been described before [5]. Briefly, at 72 h p.i. infected and mock-infected extracts were harvested and stored at −80°C. Frozen cell extracts were prepared by solubilisation in 10 volumes buffer A (20 mM Hepes pH 7.9, 10 mM KCl, 1.5 mM MgCl_2_) with protease inhibitors (Complete) and allowed to swell for 20 min at 4°C. After sedimentation (1.000 *g*, 4°C, 10 min) the cells were resuspended in 4 volumes buffer A with protease inhibitors and lysed with 30 strokes in a Dounce homogenizer (B pestle). Nuclei were sedimented and resuspended in 1.5 volumes buffer C (20 mM Hepes pH 7.9, 1.5 mM MgCl_2_, 0.42 mM NaCl, 20% (v/v) glycerol) with the same protease inhibitors as for buffer A. The salt extraction was performed for 30 min on ice. After centrifugation, the supernatant was dialyzed against DNA-binding buffer (50 mM Tris-HCl pH 8.0, 10% glycerol, 1 mM DTT, 1 mM EDTA), followed by centrifugation of insoluble material. The nuclei were frozen in aliquots at −80°C.

### DNA Binding Assay

*In vitro* DNA binding analysis was performed as described before [11]. Briefly, DNA-cellulose (Sigma-Aldrich) or matrix material from HiTrapTM Heparin columns (GE Healthcare) were loaded with [^35^S]-labeled pUL77, MCP, HSV-1 ICP8 or pUL56 diluted 1∶10 in DNA binding buffer (DBB; 50 mM ‘Tris-HCl, pH 8.0, 10% glycerol, 1 mM DTT, 1 mM EDTA). Bound protein was eluted in 0.5 ml fractions of DBB with increasing concentrations of NaCl (200, 400, 800, 1500 and 2000 mM). Fractions were subjected to 8% SDS-PAGE prior to autoradiography.

### Sequence specific binding to ds oligonucleotides

This assay (Fig. S2) is modified from the DNA binding assay described above.

50 µl Avidin resin (Thermo Scientific, Dreieich, Germany) was equilibrated in 250 µl PBS +1% Nonidet P-40 for 2 h at room temperature. In the following 5′-end biotinylated oligonucleotides (1 µg/ml) and their complementary strand (1 µg/ml; Invitrogen) were purified, combined, denatured at 95°C for 2 min and annealed at 55°C for 30 minutes.

5′-ATTTCACCCCCCCGCTAAAAACACCCCCCCGCCCAC-3′ bio-pac1 for and

5′-GTGGGCGGGGGGGTGTTTTTAGCGGGGGGGTGAAAT-3′ pac1 rev yielded the double stranded 36-mer oligonucleotide bio-pac1. In addition, a 250-, 500- and 1000-mer biotinylated DNA fragment containing pac1 motif were obtained by PCR prior to gel purification (PureLink Quick Gel Extraction Kit; Invitrogen) using pUC-aseq [7] as a template and the following primer:

5′-CGTGCACACAGCCCAGC-3′ bio-250 bp,

5′-CTACCAGCGGTGGTTTGTTTG-3′ bio-500 bp and

5′-AATTAATAGACTGGATGGAGGCGG-3′ bio-1000 bp.

5′-ATTTCACCCCCCCGCTAAAAACTCCGCCCCCCTGACGAG-3′ as reverse primer for all three amplifications.

Furthermore, a specific HCMV 966–mer biotinylated oligonucleotide encoding a region between the L/S-junction were obtained by PCR using plasmid pON205 (kindly provided by E.S. Mocarski) with the following primer:

5′- CTGGTACCGGCGCGCCCAAAAAG- 3′ bio-HCMV and

5′- CTCGAGCGCTGTCATCTAGGTG-3′ HCMV reverse primer

The bio-250 bp, bio-500 bp, bio-1000 bp and bio-HCMV double-stranded oligonucleotides were only used for analysis with pUL77, while the 36-mer bio-pac1 was used for pUL77 as well as for pUL56 and MCP. The biotinylated double-stranded oligonucleotide was bound to the avidin resin by incubation for 30 min at room temperature. For removing excess unbound oligonucleotides the resin was washed twice with PBS +1% Nonidet P-40. The resin was dissolved in 100 µl PBS +1% Nonidet P-40, 20 µl of *in vitro* translated protein pUL77, pUL56 or MCP (see above) were added and incubated at 4°C over night. After additional washing steps (50 mM Tris-HCl pH 8.8, 10% glycerol, 1 mM DTT, 1 mM EDTA, 150 mM NaCl) bound DNA-protein complexes were eluted by a sodium chloride gradient (200 mM-2000 mM NaCl) prior to boiling in SDS sample buffer. Samples were analyzed by 8% SDS-PAGE followed by autoradiography. Radioactive signals were analyzed using BioImager (Fuji, Raytest, Straubenhardt, Germany) and quantified with the image processing program ImageJ 1.41o (National Institute of Health, USA).

### Chemical cross-linking

Either purified rpUl77 or *in vitro* translated, radiolabeled pUL77 or pUL77ΔCCM were incubated 5 min at room temperature in PBS in a final volume of 20 µl. The cross-linking reaction was started by addition of 0.1% glutaraldehyde to a final concentration of 0.01%. The reaction was stopped by addition of 10 µl sodium dodecyl sulfate (SDS) loading buffer and denaturation of the samples for 2 min at 95°C. The proteins were separated by 8% SDS polyacrylamide gel electrophoresis (PAGE) and analyzed by autoradiography.

### In vitro binding assay

For *in vitro* binding analysis, equal amounts of GST fusion proteins pUL77-GST (see above) loaded on glutathione-sepharose 4B (Amersham Bioscience) were incubated with *in vitro* translated pUL77 or pUL77ΔCCM for 2 h at 4°C in 500 µl binding buffer (0,05% Nonidet P-40; 50 mM Hepes, 10% glycerol; 0,1% BSA; 300 mM NaCl; pH 7,3). Samples were washed with binding buffer and subsequently subjected to SDS-PAGE, consecutive fixation and autoradiography. The autoradiogram was quantitatively analysed using image processing software ImageJ 1.41o (National Institute of Health, USA).

### Sequence analysis

For secondary structure prediction PSIPRED (http://bioinf4.cs.ucl.ac.uk:3000/psipred) was used. For determination of putative oligomerization domains (coiled-coil motif (CCM) analysis) the following prediction programs were used:

PCOILS (http://toolkit.tuebingen.mpg.de/pcoils),

NPS (http://npsa-pbil.ibcp.fr/cgi-bin/secpred_consensus.pl),

COILS (http://www.russelllab.org/cgi-bin/coils/coils-svr.pl),

MATCHER (http://cis.poly.edu/~jps/matcher.html) and

Marcoil (http://www.isrec.isb-sib.ch/cgi-bin/BCF/webmarcoil/webmarcoilC1.cgi).

## Results

### Identification of the protein UL77

**Figure 2:**
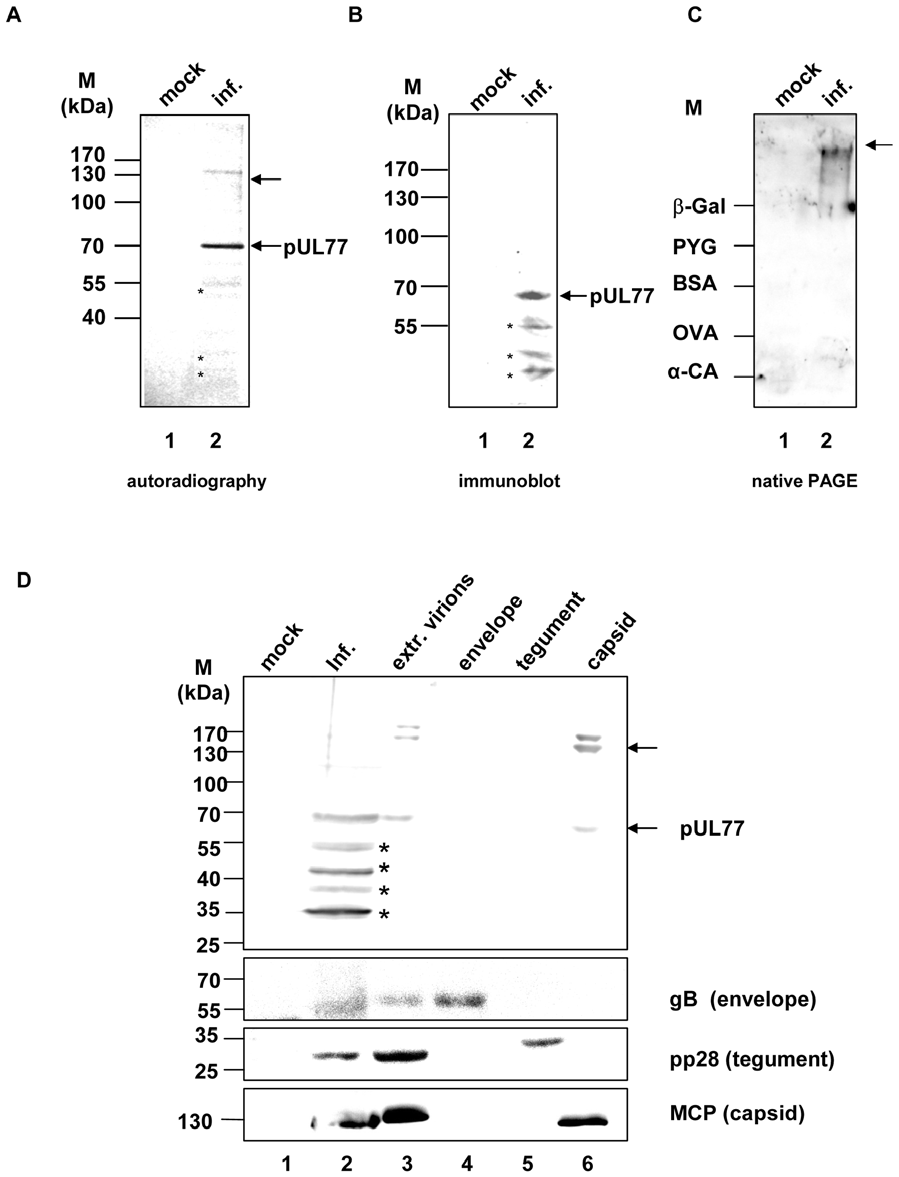
Identification of the UL77 gene product. (A) HCMV infected (lane 2) or mock-infected cells (lane 1) were 60 h p.i. radiolabeled with 50 µCi/ml [^35^S]methionine for 12 h. Cells were harvested and subjected to immunoprecipitation with pAbUL77 prior to 8% SDS-PAGE and autoradiography. (B) Extracts from mock-infected (lane 1) or HCMV-infected cells (lane 2) were used for immunoblot analyses with antibody pAbUL77 prior to 8% SDS-PAGE. The asterisks (*) indicates degradation products. Arrows on the right side indicate the positions of the monomer of pUL77 and an oligomer, while the molecular weight markers (M) are indicated on the left. (C) Extracts from mock-infected (lane 1) or HCMV-infected cells (lane 2) were subjected to 8% native gel electrophoresis followed by immunoblot analysis with pAbUL77. Marker proteins were α-carbonic anhydrase (α-CA, 29 kDa), ovalbumin (OVA; 45 kDa), BSA (66 kDa), phosphorylase b (PYG; 97 kDa) and ß-galactosidase (ß-Gal; 116 kDa). D) Association of pUL77 with viral capsids. (A) Mock- (lane 1) and infected cell extracts (lane 2) as well as extracellular virions (lane 3), envelope (lane 4), tegument (lane 5) and purified capsid (lane 6) fractions were analyzed by immunoblot with pAbUL77 and, antibodies against MCP (capsid), pp28 (tegument) or gB (envelope). The asterisks (*) indicates degradation products. The molecular weight standards (M) are indicated on the left side, the proteins are indicated by arrows.

In order to identify the protein UL77, HFF cells (2×10^6^) infected or mock-infected with HCMV AD169 (MOI 1) were radiolabeled with 50 µCi/ml [^35^S]methionine at 60 h p.i. for 12 h and subjected to immunoprecipitation with pUL77 specific pAbUL77 (Fig. 2A). In AD169 infected cell extracts two proteins, a prominent one representing the 70 kDa monomer and a faint one of approximately 140 kDa, were precipitated while no protein was detected in the control (Fig. 2A). The molecular mass of approximately 70 kDa corresponds to the calculated mass of pUL77 (71 kDa) whereas the high molecular mass form might represent an oligomer of pUL77.

To further characterize pUL77 extracts of HCMV AD169 infected or mock-infected HFF cells were subjected to SDS-PAGE prior to immunoblot analysis. Interestingly, only a protein with a size of a pUL77 monomer was recognized by the monospecific antibody pAbUL77 (Fig. 2B), which may be due to a lower sensitivity of immunostaining. Identical extracts were subjected to native PAGE followed by immunoblot analysis with pAbUL77. Under these conditions only one higher molecular weight protein was detected, thus indicating that the monomer and the oligomer are transcribed from UL77 (Fig. 2C).

### Association of pUL77 with capsids

To determine whether pUL77 is a structural component of virions the three virion fractions (i) envelope, (ii) tegument and (iii) capsid were separated by ultracentrifugation. Virions were purified from the supernatant of HCMV AD169 (MOI 3) infected HFF by sedimentation. Purification of fractions was analyzed by immunoblots using antibodies against the major capsid protein MCP, tegument protein pp28 or glycoprotein B (Fig. 2D). MCP served as a control of equal protein amounts in virion and capsid fractions as well as an indicator for the purification of the capsid fraction (Fig. 2D, lanes 3, 6). Monomeric and oligomeric forms of pUL77 separated with the capsid fraction (Fig. 2D, lane 6). The control protein gB indicating envelope fraction was detected both in virion and envelope fraction (Fig. 2D, lanes 3–4). Further indicating purity of fractions pp28 was detected only in the virion and tegument fraction (Fig. 2D, lanes 3, 5). These results suggest that HCMV pUL77 is associated with the capsid.

### Discovery of CCMs in pUL77

**Figure 3:**
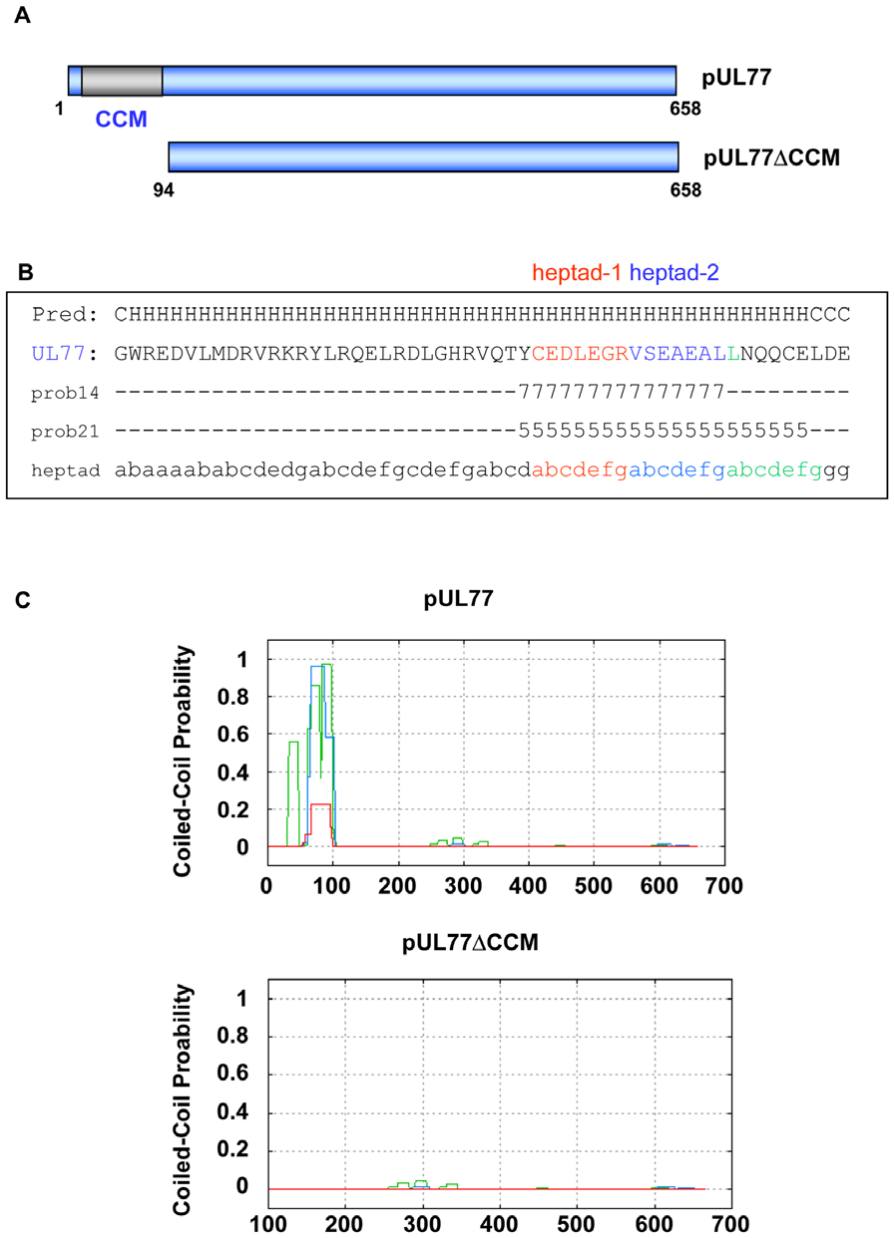
Coiled-coil motifs and secondary structure predictions of pUL77. (A) Schematically drawing of wild type pUL77 and N-terminally truncated pUL77ΔCCM, both used in this study. Position of CCM between aa 47 and aa 100 is indicated by the grey box. (B) pUL77 sequence between aa 47 and aa 100. The heptad frame (abcdefg) for scoring the CCM probabilities are shown underneath, while the secondary structure prediction by PSIPRED (http://bioinf4.cs.ucl.ac.uk:3000/psipred) is shown above the aa of pUL77. Probability scores (9 is 100%) in the scanning window 14 and 21, in the region between aa 47 and aa 100 are shown below the sequence. The first two heptads (red and blue) are predicted in both scanning windows. (C) Probability blot for the CCMs predicted by PCOILS (http://toolkit.tuebingen.mpg.de/pcoils). Probability scores on a scale of 0 to 1 (1 is 100%) are plotted against aa residue number of pUL77 and pUL77ΔCCM. Peaks in the figure indicate regions of higher coiled coil probability. MTIDK scoring matrix with weighted options was used. The default output of probabilities in the scanning windows of 14, 21 and 28 aa residues are shown in green, blue and red. One region between aa 77–91 is predicted to form coiled-coil structure. The inset shows a schematic drawing of the pUL77 protein.

In order to analyze the ability of pUL77 to form oligomers *in silico* analysis were performed. The presence of coiled-coil motifs (CCM) in the amino acid sequence was confirmed with the statistical analysis program PCOILS (Fig. 3A) using the weighting option and the profile matrix MTIDK. Coiled-coil structures are built by at least two alpha-helices that dimerize forming a supercoil [27] CCMs consist of regular alternation of three or four heptad repeat elements. The individual positions of the amino acids (aa) are labelled a–g, whereas a and d represents hydrophobic aa, e and g polar or charged aa. The probability of pUL77 showed for all scanning windows one region at the N-terminus that can form coiled-coil structures (Fig. 3C, upper panel). In this region at least three heptads were predicted in scanning window-14 and two in scanning window-21 (Fig. 3B, red, blue and green label) In addition, secondary structure analysis by PSIPRED revealed an alpha helix along this region. Even though the prediction of CCMs were done by using the to date most reliable program PCOILS [28], additional analysis were performed with four other programs. Predictions from the NPS server were identical while the COILS, MATCHER, Marcoil programs resulted in a moderate prediction (Fig. S1). These results indicated that 100 amino acids of the N-terminus of pUL77 have the ability to form coiled-coil structures.

In contrast to wild type pUL77 the N-terminally truncated pUL77ΔCCM (Fig. 3A) shows no probability to form CCM as predicted by PCOILS (Fig. 3C, lower panel). This missing coiled-coil motif indicates that this mutant might loss the ability to oligomerize. This hypothesis was tested in the following *in vitro* experiments.

### Influence of CCM deletion on oligomerization

**Figure 4:**
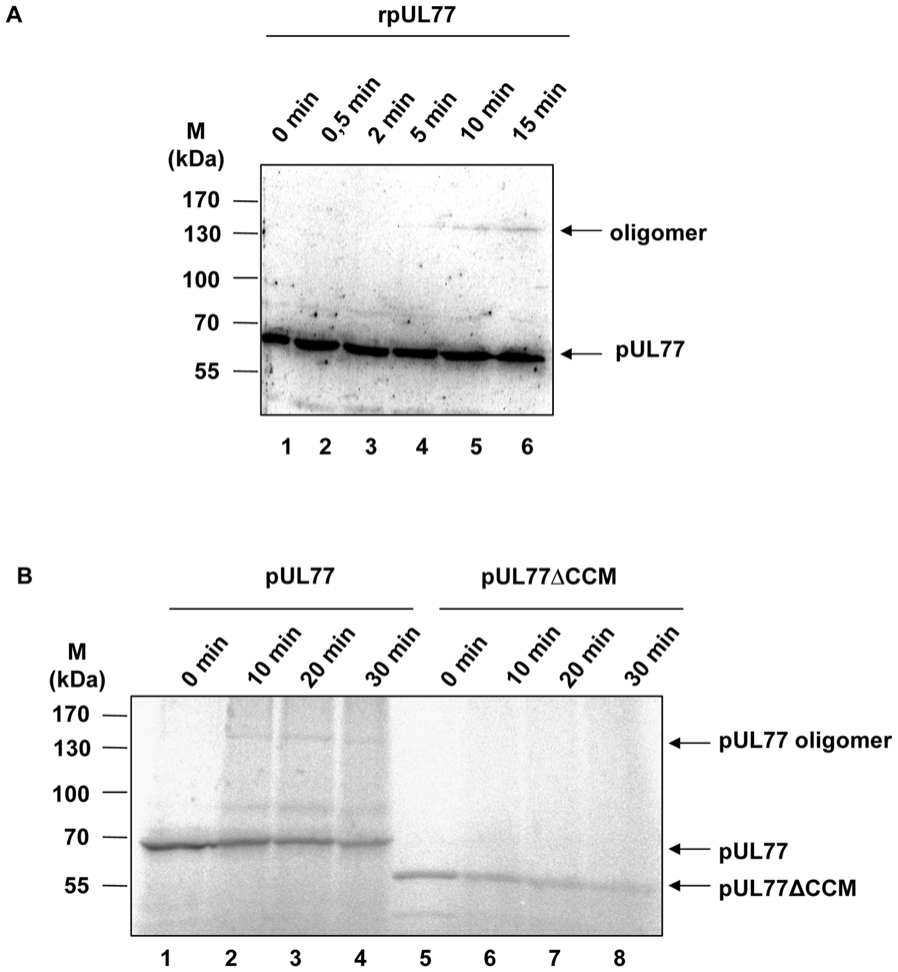
Influence of CCM deletion on dimerization. Recombinant expressed and purified (A) rpUL77 as well as *in vitro* translated (B) pUL77 or pUL77ΔCCM was reacted with glutaraldehyde for the indicated times, followed by 8% SDS PAGE and immunoblot analysis. Molecular weight markers (M) are indicated on the left side; position of the protein is indicated on the right.

To determine the ability to form oligomers purified protein rpUL77 expressed from baculovirus was examined by chemical cross-linking. The protein was treated for 0, 0.5, 2, 5, 10 and 15 min with glutaraldehyde prior to separation by SDS PAGE and detection via immunostaining. Cross-linking of rpUL77 resulted in electophoretic mobility shift, indicating the formation of dimers after approximately 10 min of incubation on ice (Fig. 4A, lane 5). In comparison, using the same approach with pUL104, dimers as well as multimers could be detected [29]. This result showed that pUL77 is able to assemble into dimers without the aid of additional viral proteins.

To investigate the ability of dimer formation further, *in vitro* translated, radiolabeled pUL77 or pUL77ΔCCM were examined by the identical cross-linking procedure. After treatment with glutaraldehyde and separation by SDS PAGE the detection was performed by autoradiography. Cross-linking of wild-type pUL77 (Fig. 4B lane 1–4) leads to a band of higher molecular weight after 10 min glutaraldehyde treatment, which corresponds to the size of a dimer, whereas no oligomer was observed in probes containing the mutant pUL77ΔCCM (Fig. 4B lane 5–8). Taking together these two results suggest that the coiled-coil domain is a prerequisite for the ability of pUL77 to form homodimers.

### Interaction of HCMV pUL77 with DNA packaging proteins

**Figure 5:**
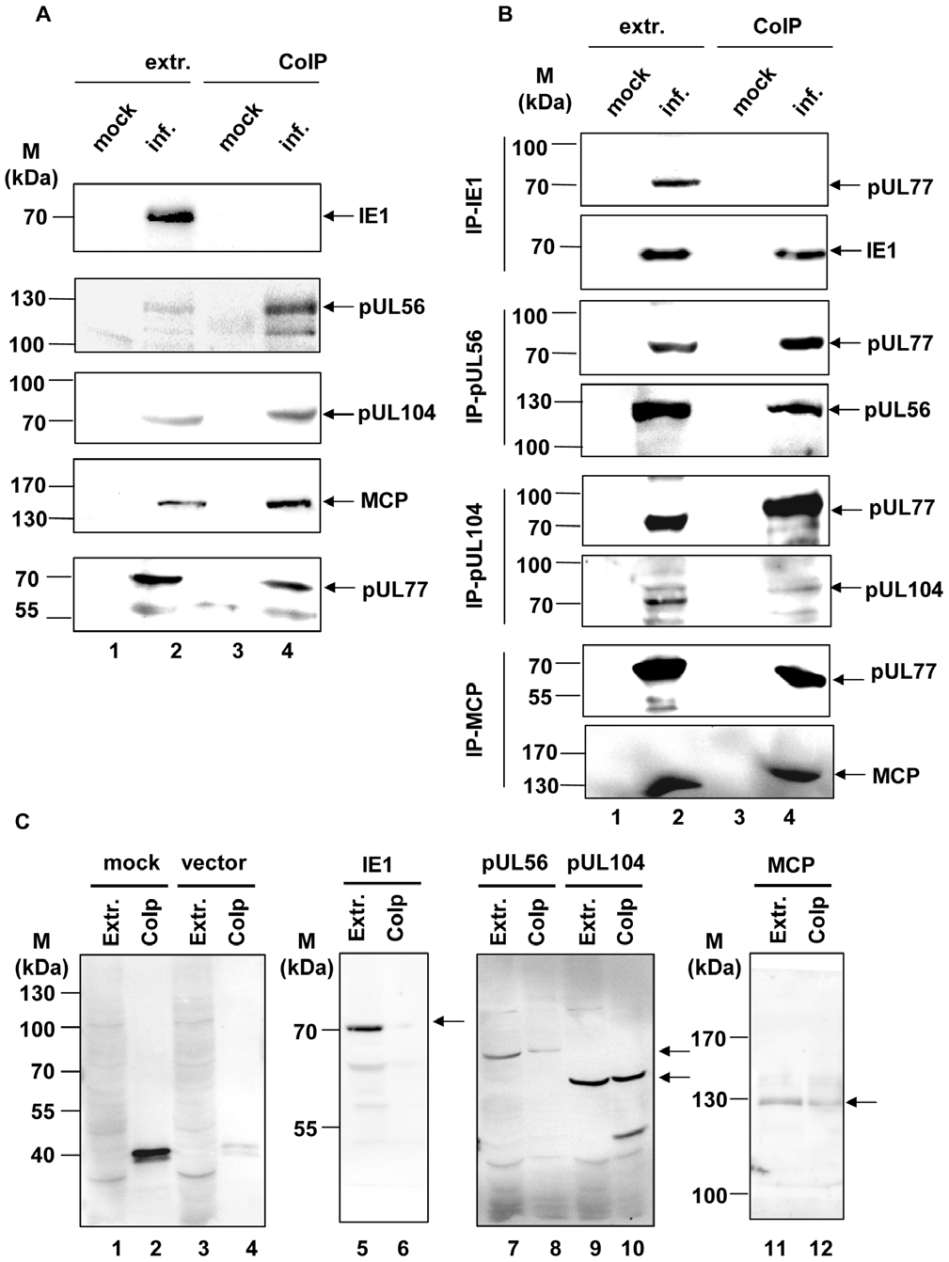
Interaction to pUL77 with DNA packaging proteins. (A) Mock-infected or HCMV-infected HFF lysates at 72 h p.i. were co-immunoprecipitated with pAbUL77 and subjected to SDS-PAGE prior to transfer onto nitrocellulose. Immunostaining was performed using antibodies against IE1, pUL56, MCP, pUL104 and pUL77. (B) Mock-infected or HCMV-infected HFF lysates at 72 h p.i. were co-immunoprecipitated with specific antibodies against IE1, pUL56, MCP and pUL104 and subjected to SDS-PAGE prior to transfer onto nitrocellulose. Immunostaining was performed using pAbUL77 and as a control antibodies used for precipitation. (C) 293T cells were co-transfected with myc-UL77 and pcDNA3.1/HisC (lanes 3–4), myc-UL77 and pHM123 encoding IE1 (lanes 5–6), myc-UL77 and pcDNA-UL56 (lanes 7–8), myc-UL77 and pcDNA-UL104 (lanes 9–10). or myc-UL77 and pcDNA-MCP (lanes 11–12). 48 h after transfection co-immunoprecipitation was performed with specific mAb Myc-Tag and subjected to SDS-PAGE prior to transfer onto nitrocellulose. Immunoblot analyses using specific mAb Anti-Xpress tag of pcDNA-UL56, pcDNA-UL104 and pcDNA-MCP or using mAb3-27 for pHM123 was performed. The arrows indicate the positions of the proteins IE1, pUL56, MCP, pUL104 and pUL77 The molecular mass markers (M) are indicated on the left.

In order to investigate direct interactions between pUL77 and capsid-associated proteins analysis in the viral context were performed using mock-infected or HCMV AD169 (MOI 2) infected cells. For co-immunoprecipitations either the monospecific antibody against pUL77 (Fig. 5A) or against IE1, pUL56, pUL104 and MCP (Fig. 5B) were used. The following immunostaining detected pUL56 as the full size protein of 130 kDa (Fig. 5A, pUL56), MCP at 145 kDa (Fig. 5A, MCP), the monomer of pUL104 at 70 kDa (Fig. 5A, pUL104) and pUL77 to analyze the presence of both precipitated proteins (Fig. 5A, pUL77) in HCMV-infected cell extracts and co-immunoprecipitates. No protein was detected in mock-infected cell extracts or co-immunoprecipitates (Fig. 5A, lanes 1, 3). Immunostaining against IE1 was used as a negative control (Fig. 5B, lane 4, IE1). In addition, precipitations using antibodies specific for pUL56, pUL104, MCP and IE1 detected pUL77 in the corresponding manner, thus confirming the interactions (Fig. 5B).

To further examine whether interactions between pUL77 and packaging proteins are obtained with solitary expressed proteins co-immunoprecipitation in 293T-transfected cells were performed. For precipitation Myc-tag antibody prior to immunostaining against Xpress tag of IE1, pUL56, pUL104 and MCP was used. In precipitates monomers of pUL56, pUL104 and MCP were detected (Fig. 5C, lanes 8, 10, 12). In precipitates of vector-transfected cell extracts as well as IE1-transfected cells no specific proteins were observed (Fig. 5C, lane 4, 6).

These data implicate physical interactions between the capsid-associated pUL77 and the structural proteins of the HCMV capsid (i) the large terminase subunit pUL56, (ii) the portal protein pUL104 and (iii) MCP.

### DNA binding of HCMV pUL77

**Figure 6:**
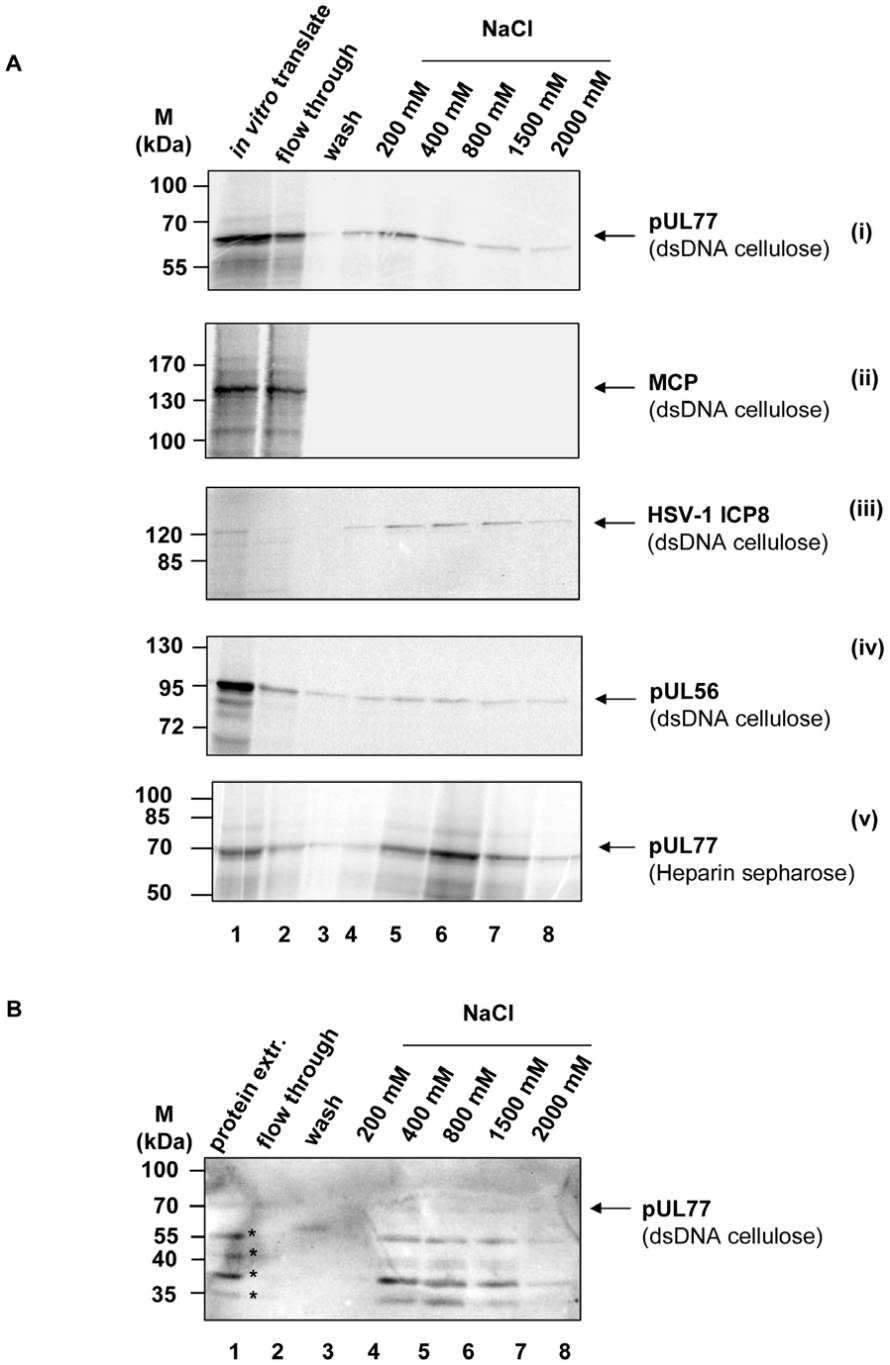
Interaction of HCMV pUL77 with DNA. (A) *In vitro* translated pUL77 (i), MCP (ii), HSV-1 ICP8 (iii), or pUL56 (iv) (lane 1) were applied to a dsDNA cellulose column. After the column was washed, DNA-binding proteins were eluted with increasing NaCl concentrations (lanes 3–8). To verify the DNA binding ability of pUL77 the protein was loaded onto a heparin column. The elution was performed as described above (3–8). Nuclear extracts of infected HFF were applied to the dsDNA cellulose (lane1). Elution was performed with increasing NaCl concentrations as described above (lanes 3–8). For detection of pUL77 immunoblot analysis using the monospecific pAbUL77 was performed. The asterisks (*) indicates degradation products. Molecular mass markers (M) are indicated on the left; the positions of the proteins are indicated on the right.

To determine the ability of pUL77 to bind dsDNA, *in vitro* binding studies were performed. Radiolabeled pUL77 (Fig. 6A (i)) or MCP as a negative control (Fig. 6A (ii)) or HSV-1 ICP8 (Fig. 6A (iii) and pUL56 (Fig. 6A (iv)) as positive controls were loaded on a dsDNA cellulose column. After the washing steps bound proteins were eluted with a sodium chloride gradient. Eluates were analyzed by SDS-PAGE and autoradiography. pUL77 as well as both positive controls pUL56 and ICP8 could be detected in the eluted fractions (Fig. 6A (iii–iv)) thus implying an interaction with dsDNA. In contrast to this observation there was no significant signal for MCP (Fig. 6A (ii)). In addition, binding of pUL77 to heparin was analysed, because its negative charge mimics the electrostatic characteristics of dsDNA and therefore heparin-sepharose is used to bind DNA binding proteins. *In vitro* translated pUL77 was loaded on a heparin sepharose and eluted as described above. The protein was detected in the eluted fraction (Fig. 6A (v)). To determine whether pUL77 from infected cells binds to DNA, nuclear extracts were prepared for dsDNA cellulose binding assay. A band representing pUL77 as well as a degradation product were detected in the eluted fractions by the monospecific antibody (Fig. 6B). These results demonstrated that pUL77 can bind to dsDNA.

### HCMV pUL77 binds to dsDNA with sizes of at least 500 bp

**Figure 7:**
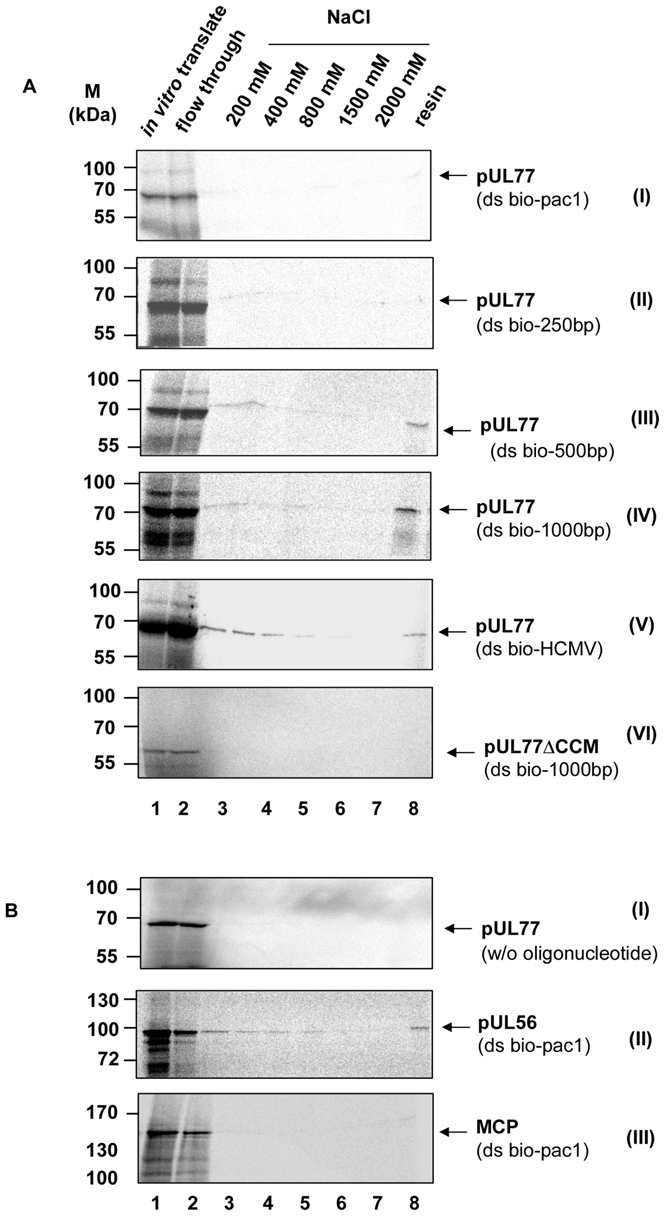
HCMV pUL77 binds to specific DNA. (A) The binding reaction was carried out by incubation of dsDNA oligonucleotides 36-mers containing pac1 motif ((I), lanes 1–8), 250-mer ((II), lanes 1–8), 500-mer ((III), lanes 1–8), 1000-mer ((IV), lanes 1–8) and HCMV-specific 966-mer ((V), lanes 1–8) with avidin agarose resin followed by addition of *in vitro* translated pUL77 or pUL77ΔCCM to 1000-mer ((VI) lane 1–8). (B) In order to control unspecific binding abilities of protein to bead material pUL77 was incubated with avidin beads only ((I) lane 1–8). In addition, the specificity of the method was investigated with known interactions. To the 36-mers containing pac1 motif (lanes 1–8) on avidin beads *in vitro* translated pUL56 ((II), lane 1) or MCP ((III)l, lane 1) were added as positive and negative control. The resin was subjected to serious washing steps (lanes 2–7). DNA-binding proteins were eluted by boiling in SDS sample buffer (lanes 8). Samples were analyzed by 8% SDS-PAGE and autoradiography. Molecular mass standards are shown to the left, the positions of the proteins are indicated on the right.

To analyze whether pUL77 has the ability to bind to the pac 1 motif or certain DNA fragments, double-stranded 36-mer oligonucleotides as well as 250, 500, 1000 bp and a HCMV-specific 966 bp (bio-HCMV) fragments with incorporated biotin were bound to avidin agarose resin prior to incubation with *in vitro* translated, radiolabelled pUL77 (Fig. 7A, (I)–(V)) or pUL77ΔCCM (Fig. 7A, (VI)), pUL56 (Fig. 7B, lane (II)) or MCP (Fig. 7B, (III)). The resin was subjected to sequential washing steps prior and after binding of *in vitro* translated proteins and elution steps (Fig. 7, lanes 2–8). Thus, pac 1 motif alone is not sufficient for binding (Fig. 7A (I)) nor oligonucleotides smaller than 500 bp (Fig. 7A (II)). However, binding of pUL77 was observed if the oligonucleotides bio-500 bp, bio-1000 bp DNA or bio-HCMV were used (Fig. 7A (III)–(V)). In order to interpret the findings intensity of signals were quantitatively analysed. The binding efficiency (BE) determined by image processing software was 2.2±0.41 for bio-500 bp and 4.9±0.72 for bio-1000 bp, respectively (Table 1). In contrast, the terminase subunit pUL56 showed a sequence specific binding to bio-pac1 with a BE of 10.0±0.63 (Fig. 7B (II), lane 8; Table 1). By using the negative control MCP no signal was detected (Fig. 7B (III), lane 8; Table 1). No binding was observed using pUL77ΔCCM (Fig. 7A (VI)). The complete deletion of the CCM negatively influences pUL77-associated DNA binding to biotinylated 1000 bp dsDNA. These data indicates that pUL77 is able to bind to dsDNA with a size of at least 500 bp or longer in a non pac 1 motif specific manner.

**Table 1:** Binding efficiency to double-stranded oligonucleotides.

Protein	dsOligonucleotide	Binding Efficiency (BE)*
pUL56	bio-pac1	10.0±0.63
MCP	bio-pac1	0.2±0.40
pUL77	bio-pac1	0.2±0.03
pUL77	bio-250 bp	0.5±0.45
pUL77	bio-500 bp	2.2±0.41
pUL77	bio-1000 bp	4.9±0.72

*BE = binding to resin^§^/*in vitro* tanslate^§^×100; BE = 1 = positive ^§^adjusted to 100%.

Values were obtained from three independent experiments.

## Discussion

Viral packaging and subsequent cleavage of concatemeric DNA into genome units are multifunctional steps in which several gene products are involved. In the case of HSV-1 it is known that at least seven proteins, the gene products of UL6 [30]–[33], UL15 [34], [35], UL17 [36], [37], UL25 [38], [39], UL28 [34], [40], UL32 [41] and UL33 [42], are required for DNA cleavage and packaging. Homologous proteins are found in HCMV, but the mode of action of each protein is not known to date and has to be elucidated.

In the present study the HCMV protein UL77 (pUL77) [43], the structural homolog to HSV-1 and PrV pUL25, which is required for viral replication, was characterized. Analyses were performed with an affinity-purified antibody against pUL77. In HCMV infected cells pUL77 has a molecular mass of approximately 70 kDa with a tendency to form oligomers. In infected cells the observed oligomer could be a heterodimer of pUL77 and pUL93, the homologue of pUL17 of HSV-1 [36], [37]. However, our results with purified solitary expressed rpUL77 as well as *in vitro* translated protein implicates that pUL77 has the ability to form a homodimer. Our data are in line of the recently reported data from To et al [44] demonstrating by yeast two hybrid analysis and co-immunoprecipitation that pUL77 could form homodimers.

Even though pUL77 is not a glycoprotein as hypothesized earlier [45], the oligomer formation in the virus could possible be mediated via disulfide bonds. Since the oligomer was observed under denaturing/reducing conditions on a SDS-polyacrylamide gel it is more likely that the oligomer formation occurs by a covalent binding. Interestingly, Bowman et al. suggested that the N-terminal 133 residues of HSV-1 homolog pUL25 could oligomerize via a coiled-coil interaction [46]. Sequence analysis using the statistical program PCOILS predicted the presence of a coiled-coil motif (CCM) in the first 100 amino acids of pUL77. Experiments with a CCM deletion mutant of pUL77 show that oligomerization was no longer observed, thus indicating that CCM is required for oligomer formation. The CCM is an oligomerization motif for many proteins [27] including motor proteins, cytoskeleton proteins and transcription regulators. All of these proteins form coiled-coils for folding as well as oligomerization. These predications are consistent with our *in vitro* data. Furthermore, secondary structure analysis by PSIPRED predicted a long stretch of helices in the CCM. Helices are thought to enhance the stability of coiled-coil structures. Two of the most stable proteins are tetrabrachion, a surface protein from archeabacterial and Sendai virus phosphoprotein. Peters et al [47] reported that tetrabrachion tetramers, stabilized by CCMs, could only be denatured by 70% sulphuric acid or heating to 130°C for 30 min in 6 M guanidine hydrochloride. Regarding to our results with the pUL77 CCM mutant it is reasonable to hypothesize that the stability of the pUL77 oligomer under denaturing/reducing conditions is due to the coiled-coil structure.

The subviral localisation of pUL77 was examined by separation of the capsid from purified virions prior to immunoblot analysis. HCMV pUL77 oligomers were detected in purified extracellular virions and capsids, thus demonstrating that pUL77 is a capsid-associated structural protein. This is in agreement with several observations with the homologous gene of HSV-1 and PrV [19]–[21], [48]. More recently, it has been suggested that the protein is located at the vertexes of the capsid [17]–[19]. In addition, evidence was provided that HSV-1 pUL25 is localized on the capsid surface and might play a role in stabilization of capsids upon the increasing pressure during DNA encapsidation [19], [49], [50]. It is reasonable to speculate that this is as well a function of HCMV pUL77.

In order to verify the function of pUL77 as a capsid associated DNA packaging protein studies concerning interactions with packaging proteins were performed. Co-immunoprecitations of infected cells as well as transfected cells demonstrated that HCMV pUL77 interacts with the major capsid protein (MCP) as well as the large terminase subunit pUL56 and the portal protein pUL104. These physical interactions with the DNA packaging motor components pUL56, in contrast to all other known terminase subunits a structural component associated with the capsid, and the portal protein pUL104 supports the proposed function of pUL77 in anchoring DNA during encapsidation [4], [9], [10].

Recent findings indicate that the homologous proteins do not interfere with cleavage of concatemeric DNA but are involved in later stages of packaging like providing a plug for the encapsidated DNA [20], [37], [39]. In the case of HSV-1 it has been shown that a UL25 null virus leads to an accumulation of A-capsids that lost DNA during packaging [37]. Therefore another feature of these proteins might be an at least short-term binding of DNA. To support one or the other hypothesis it would be interesting to analyze the ultrastructural phenotype of a 77-null-mutant virus, which is currently beyond the aim of this study.

None the less, short-term DNA binding would be a prerequisite for both proposed functions. By using an *in vitro* assay we demonstrated the ability of sequence-independent DNA binding of pUL77. Our results are in line with the report of Ogasawara [50] showing that HSV-1 pUL25 binds to genomic DNA. In contrast to the terminase subunit pUL56 the binding was only observed with the 500- and 1000-meric dsDNA. One explanation of this observation could be due to the different functions of the proteins. While the sequence specific pUL56 mediated DNA-binding is required for insertion into the capsid, the association of pUL77 with DNA is more likely a temporary stabilization. In addition, one could hypothesize that the observed effect is due to the negative charge of the DNA. The enlargement of DNA fragments will result in increasing negative charges. Since the isoelectric point (pI) of pUL77 is 5.73, the protein is charged negative at the used pH 7.3. Therefore the observed effect is not unspecific due to a positive charged protein. Furthermore, the binding to Heparin column is a characteristic of DNA binding proteins. It would be interesting to identify the DNA binding site. In order to get a hint CCM analysis was performed. We demonstrated that DNA binding was no longer observed in pUL77ΔCCM mutant lacking the CCM, thus showing that oligomerization is a prerequisite for DNA binding in this case. The observed interaction with HCMV DNA implicated a potential function of pUL77 during packaging. One possible explanation could be that HCMV pUL77 plays a role in anchoring the encapsidated DNA during the late stage of packaging. To this end, further studies will need to be performed aimed at deepening our understanding concerning the role of HCMV pUL77 in viral life cycle.

## Supporting Information

Figure S1 CCM predictions in pUL77 using different programs. Predictions are rated on the basis of probability values; higher the probability, stronger the prediction: ++++, strong; +++, moderate, +, weak; −, none.
(PPT)

Figure S2 Schematic diagram of the binding to biotinylated ds oligonucleotides. (I) Biotinylated ds oligonucleotides were incubated with avidin resin. (II) Several washing steps to remove unbound DNA. (III) Addition of radiolabeled *in vitro* translated protein. (IV) Washing steps II to remove unbound protein prior to incubation at 20°C. (V) Elution via a sodium chloride gradient and heating to 95°C. (VI) Analysis by autoradiography.
(PPT)
