# Supplementary material for: A “Coiled-Coil” Motif Is Important for Oligomerization and DNA Binding Properties of Human Cytomegalovirus Protein UL77
Source: PLoS One. 2011 Oct 5;6(10):e25115. doi: 10.1371/journal.pone.0025115 (PMC3187746; doi:10.1371/journal.pone.0025115)
Supplement: Figure S1 — CCM predictions in pUL77 using different programs. Predictions are rated on the basis of probability values; higher the probability, stronger the prediction: ++++, strong; +++, moderate, +, weak; −, none. (PPT) [file pone.0025115.s001.ppt]

## Slide 1
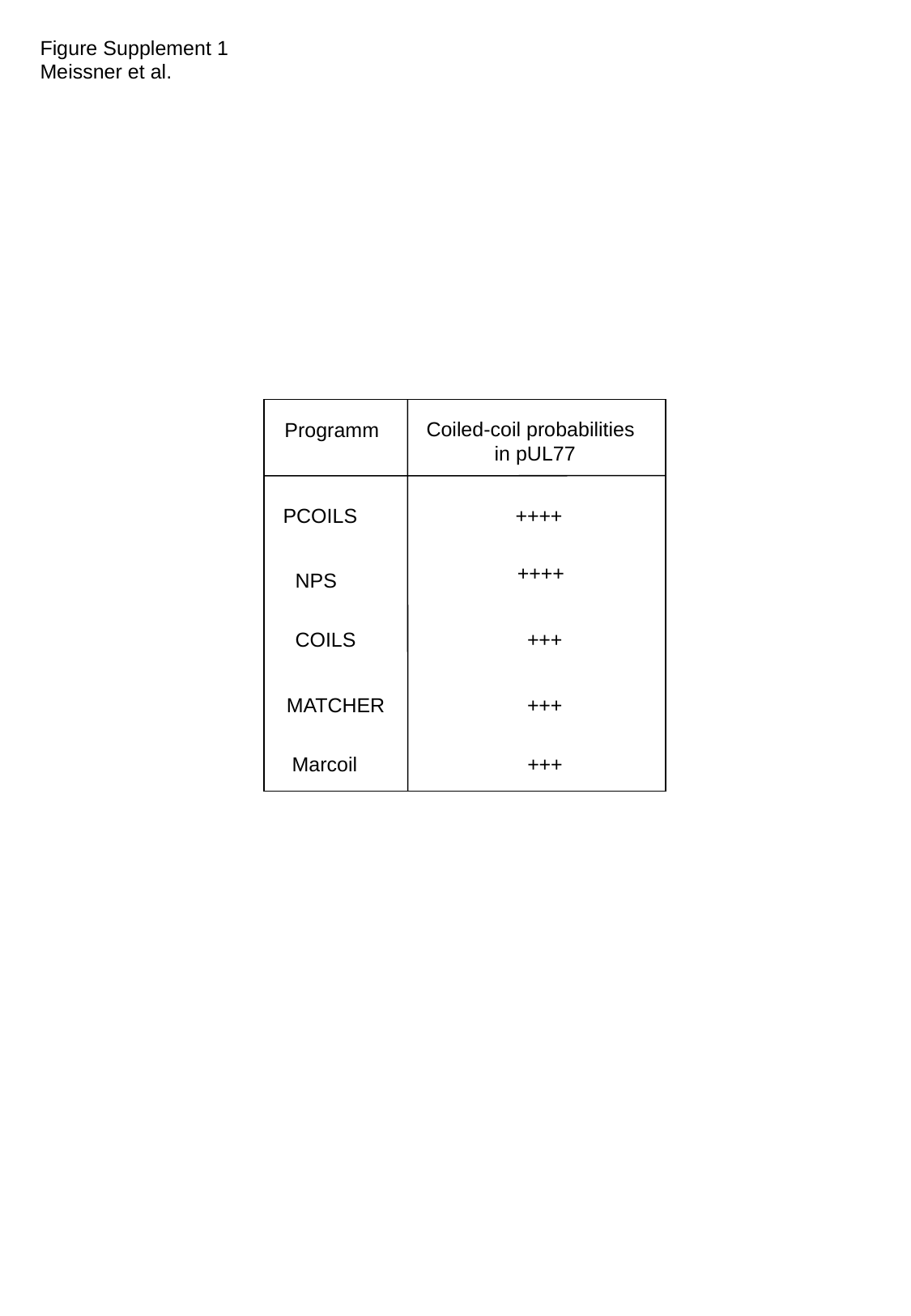

Figure Supplement 1
Meissner et al.
 Coiled-coil probabilities
 in pUL77
Programm
PCOILS
++++
++++
NPS
COILS
+++
MATCHER
+++
Marcoil
+++
