# Supplementary material for: A “Coiled-Coil” Motif Is Important for Oligomerization and DNA Binding Properties of Human Cytomegalovirus Protein UL77
Source: PLoS One. 2011 Oct 5;6(10):e25115. doi: 10.1371/journal.pone.0025115 (PMC3187746; doi:10.1371/journal.pone.0025115)
Supplement: Figure S2 — Schematic diagram of the binding to biotinylated ds oligonucleotides. (I) Biotinylated ds oligonucleotides were incubated with avidin resin. (II) Several washing steps to remove unbound DNA. (III) Addition of radiolabeled in vitro translated protein. (IV) Washing steps II to remove unbound protein prior to incubation at 20°C. (V) Elution via a sodium chloride gradient and heating to 95°C. (VI) Analysis by autoradiography. (PPT) [file pone.0025115.s002.ppt]

## Slide 1
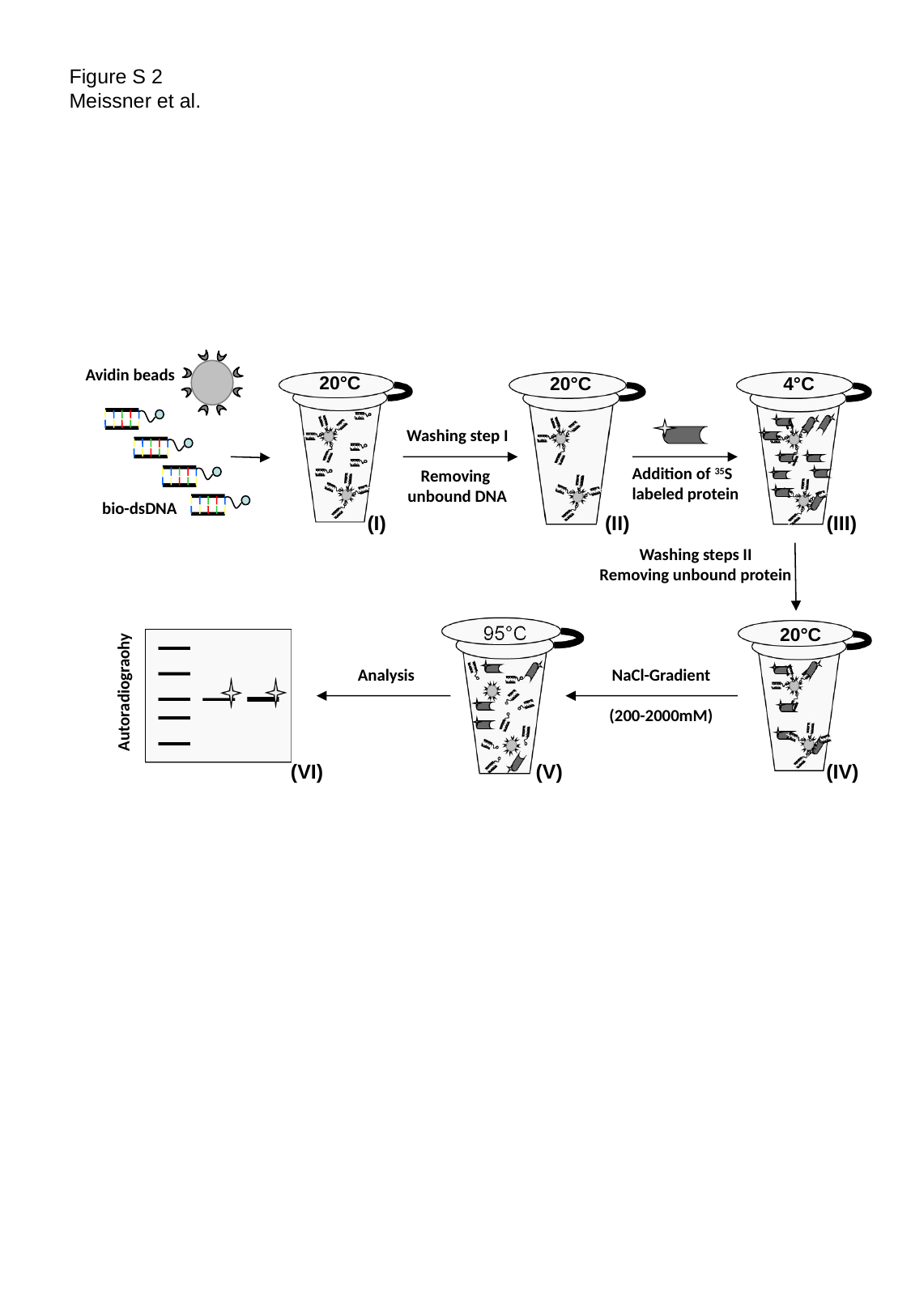

Figure S 2
Meissner et al.
Avidin beads
20°C
20°C
4°C
Washing step I
Removing
unbound DNA
Addition of 35S
labeled protein
bio-dsDNA
(I)
(II)
(III)
Washing steps II
Removing unbound protein
20°C
Analysis
NaCl-Gradient
(200-2000mM)
Autoradiograohy
(VI)
(V)
(IV)
